# Strategies to prevent, diagnose and treat kidney disease related to systemic arterial hypertension: a narrative review from the Mexican Group of Experts on Arterial Hypertension

**DOI:** 10.1186/s12882-023-03450-5

**Published:** 2024-01-18

**Authors:** Silvia Palomo-Piñón, José Manuel Enciso-Muñoz, Eduardo Meaney, Ernesto Díaz-Domínguez, David Cardona-Muller, Fabiola Pazos Pérez, Emilia Cantoral-Farfán, Juan Carlos Anda-Garay, Janet Mijangos-Chavez, Neftali Eduardo Antonio-Villa, on behalf of the Mexican Group of Experts on Arterial Hypertension, Luis Alcocer, Humberto Álvarez-López, Ernesto G. Cardona-Muñoz, Adolfo Chávez-Mendoza, Enrique Díaz-Díaz, Héctor Galván-Oseguera, Martin Rosas-Peralta, Vidal José González Coronado

**Affiliations:** 1 Grupo de Expertos en Hipertensión Arterial México (GREHTA), Ciudad de México, México; 2 Colaborador Externo, Unidad de Investigación Médica en Enfermedades Nefrológicas Siglo XXI (UIMENSXII), UMAE Hospital de Especialidades “Dr. Bernardo Sepúlveda G” Centro Médico Nacional Siglo XXI, Instituto Mexicano del Seguro Social, Ciudad de México, México; 3 Grupo Colaborativo en Hipertensión Arterial (GCHTA), Ciudad de México, México; 4 Grupo de Expertos en Hipertensión Arterial México (GREHTA), Calle Retorno del Escorial #13, Col. El Dorado, Tlanepantla de Baz, Estado de México 54020 México; 5 Asociación Mexicana para la Prevención de la Aterosclerosis y sus Complicaciones A.C, Ciudad de México, México; 6 Escuela Superior de Medicina, Instituto Politecnico Nacional, Ciudad de México, México; 7 UMAE Hospital de Cardiología, Centro Médico Nacional Siglo XXI, Instituto Mexicano del Seguro Social, Ciudad de México, México; 8 Universidad de Guadalajara, Guadalajara, Jalisco México; 9 UMAE Hospital de Especialidades “Dr. Bernardo Sepúlveda G” Centro Médico Nacional Siglo XXI, Instituto Mexicano del Seguro Social, Ciudad de México, México; 10 Jefatura de Nefrología, Hospital General De Zona Médico Familiar No. 8 Gilberto Flores Izquierdo, Instituto Mexicano del Seguro Social, Ciudad de México, México; 11 Jefatura de Cardiología, UMAE Dr. Antonio Fraga Mouret, Centro Médico Nacional La Raza, Instituto Mexicano del Seguro Social, Ciudad de México, México; 12 Departamento de Endocrinologia, Instituto Nacional de Cardiología Ignacio Chávez, Ciudad de México, México

**Keywords:** Systemic arterial hypertension, Chronic kidney disease, Hypertension treatment, Guidelines

## Abstract

This narrative review highlights strategies proposed by the Mexican Group of Experts on Arterial Hypertension endorsed to prevent, diagnose, and treat chronic kidney disease (CKD) related to systemic arterial hypertension (SAH). Given the growing prevalence of CKD in Mexico and Latin America caused by SAH, there is a need for context-specific approaches to address the effects of SAH, given the diverse population and unique challenges faced by the region. This narrative review provides clinical strategies for healthcare providers on preventing, diagnosing, and treating kidney disease related to SAH, focusing on primary prevention, early detection, evidence-based diagnostic approaches, and selecting pharmacological treatments. Key-strategies are focused on six fundamental areas: 1) Strategies to mitigate kidney disease in SAH, 2) early detection of CKD in SAH, 3) diagnosis and monitoring of SAH, 4) blood pressure targets in patients living with CKD, 5) hypertensive treatment in patients with CKD and 6) diuretics and Non-Steroidal Mineralocorticoid Receptor Inhibitors in Patients with CKD. This review aims to provide relevant strategies for the Mexican and Latin American clinical context, highlight the importance of a multidisciplinary approach to managing SAH, and the role of community-based programs in improving the quality of life for affected individuals. This position paper seeks to contribute to reducing the burden of SAH-related CKD and its complications in Mexico and Latin America.

## Introduction

**Fig. 1:**
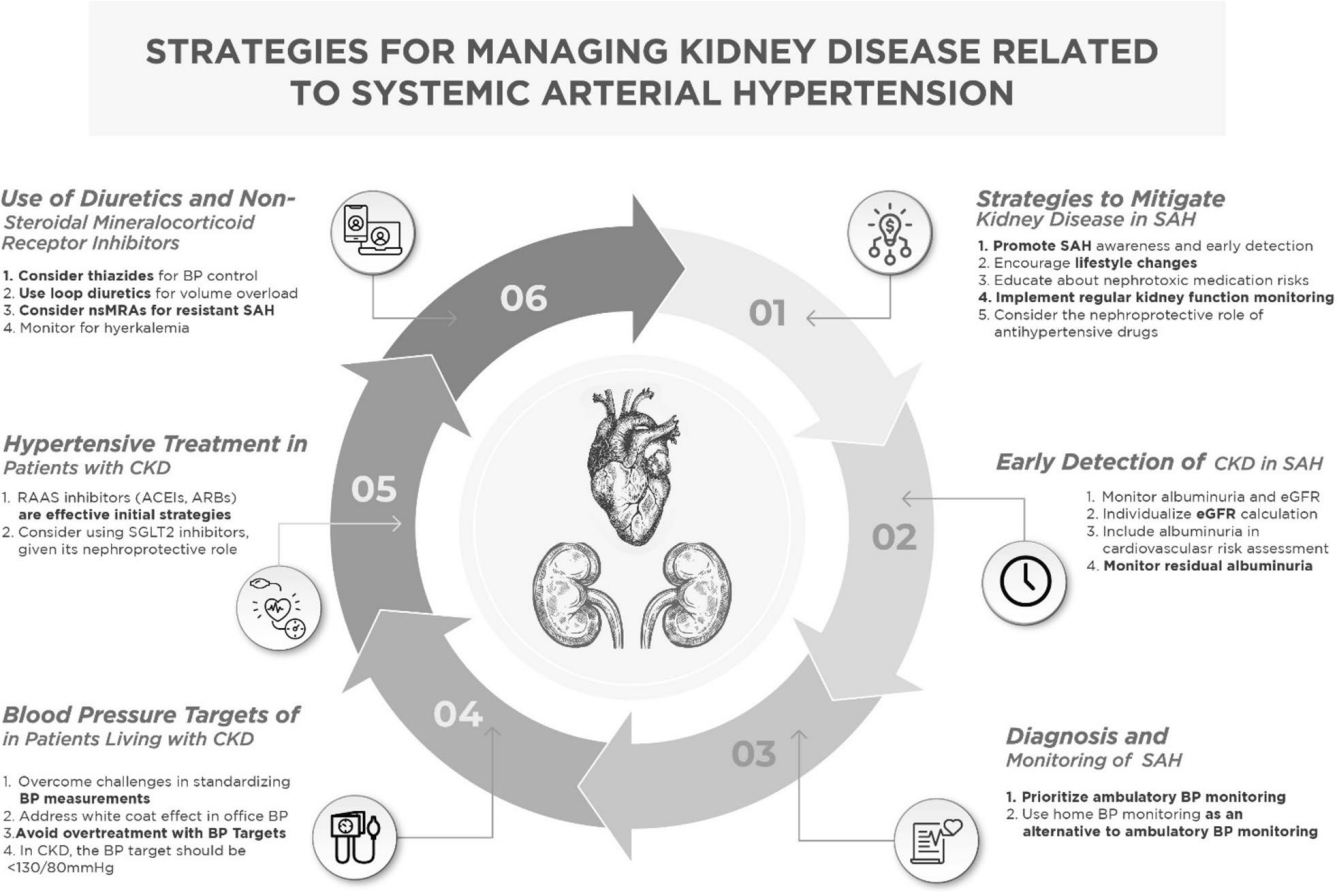
Summary of strategies for managing kidney disease related to systemic arterial hypertension in Mexico and Latin America. *Abbreviations*: SAH: Systemic Arterial Hypertension; CKD: Chronic Kidney Disease; eGFR: Estimated Glomerular Filtration Rate; BP: Blood Pressure; SGLT2: Sodium-glucose Cotransporter-2; RAAS: Renin–Angiotensin–Aldosterone System; ACEIs: Angiotensin-Converting Enzyme; ARBs: Angiotensin II Receptor Blocker; nsMRA: Non-Steroidal Mineralocorticoid Receptor Antagonists

Chronic kidney disease (CKD) is a growing public health concern globally, with an estimated prevalence of 13.4% worldwide and 10% in Latin America [1, 2]. Systemic Arterial Hypertension is a leading cause of CKD, contributing to approximately 30% of all CKD cases. In Mexico and Latin America, the burden of systemic arterial hypertension and its complications – including CKD – is substantial and increasing [3]. The escalating prevalence of CKD in the region can be attributed to multiple factors, such as an aging population, increased prevalence of diabetes and obesity, and inadequate access to healthcare services [4]. Early identification and management of systemic arterial hypertension are essential to prevent or delay the progression of kidney damage and reduce associated morbidity and mortality. It is essential to formulate context-specific approaches to address the effects of systemic arterial hypertension in Mexico and Latin America, considering the vast and diverse population of the region. These strategies should be tailored to tackle the unique challenges faced by the region, considering the socioeconomic, cultural, and healthcare system factors that lead to disparities in systemic arterial hypertension and CKD care [5]. In this narrative review, we seek to offer a comprehensive and context-specific guide for healthcare providers on the prevention, diagnosis, and treatment of kidney damage related to systemic arterial hypertension in Mexico and Latin America. For this purpose, we followed the Delphi technique to identify and address six specific topics related to primary prevention, early detection, evidence-based diagnostic approaches, and selecting pharmacological treatments [6]. By addressing topics, we aim to reduce the burden of systemic arterial hypertension-related CKD and its complications in the region. Emphasizing the importance of a multidisciplinary approach and the role of community-based programs, this review seeks to improve the quality of life for millions of affected individuals. In Fig. 1, we summarize and synthesize these strategies for clinical scenarios.

### Section 1: strategies to mitigate kidney disease

Systemic arterial hypertension and kidney disease (KD) are interlinked through complex pathophysiological pathways; these relationships position the kidney as both a target and a causative factor in hypertension. Disentangling the precise pathogenic role of moderate hypertension in kidney damage is challenging, as other risk factors or associated morbidities often coexist. In the United States, systemic arterial hypertension and impaired kidney function (glomerular filtration rate, [GFR], between 60–89 mL/min/1.73 m^2^) prevalence have been consistently increasing, reaching 28.9% and 51.2%, respectively [7]. From 1999 to 2004, the occurrence of CKD with GFR < 60 mL/min/1.73 m^2^ rose to 8%. Once KD is evident with the onset of albuminuria, the progression of CKD accelerates; this is particularly relevant when systemic arterial hypertension is accompanied by diabetes, hyperuricemia, or another KD. Considering these challenges, it is essential to create strategies to mitigate systemic arterial hypertension in patients living with CKD.

#### Question 1.1: can kidney damage and the development of CKD be prevented in patients with primary systemic arterial hypertension?

Preventing KD caused primarily by systemic arterial hypertension involves preventing its onset. The main etiological factors of systemic arterial hypertension include inherited hypertension phenotypes, epigenetic inheritance, and acquired or environmental factors. Although genetic factors cannot currently be altered, it may be possible to modify epigenetic and environmental factors related to systemic arterial hypertension onset [4]. Overall, lifestyle and dietary changes, along with the effects of specific drugs, contribute to lowering blood pressure levels and promote nephroprotection, which may hinder or delay systemic arterial hypertension onset in some individuals [8]. A second measure consists in providing adequate control of blood pressure levels in patients with systemic arterial hypertension to reduce macro- and micro-vascular complications associated with systemic arterial hypertension. In this regard, choosing and monitoring antihypertensive medications is crucial to reducing systemic arterial hypertension-associated complications. In the context of Mexico and Latin America, the following prevention measures are endorsed for both the general population and susceptible individuals:

1. *National information campaigns and increased physician awareness:* Early detection of systemic arterial hypertension is essential for preventing systemic arterial hypertension-related kidney damage. National campaigns should raise awareness regarding abdominal obesity, which has been demonstrated to be a main contributor to the development of cardiometabolic diseases, including systemic arterial hypertension [9]. Additionally, all healthcare personnel should prioritize systemic arterial hypertension awareness and detection for the general population, from medical schools to practicing doctors, including internists, endocrinologists, neurologists, cardiologists, and nephrologists.
2. *Behavioral lifestyle modifications for patients living with systemic arterial hypertension:* Three interventions have been proven to reduce the risk of systemic arterial hypertension-related outcomes: the DASH diet, reducing salt intake to < 5 g/day (2 g of sodium), and weight loss. These three interventions lower blood pressure in both normotensive and hypertensive individuals, preventing kidney damage onset and progression. Additionally, it is important to warn against the potential harm of high-protein diets and supplements, as intake of > 0.8 g of protein/kg of ideal weight, along with obesity and excess salt consumption, can cause hyperfiltration and glomerular hypertension [10–12].
3. *Caution regarding nephrotoxic agents:* Physicians should aim to educate the general population and susceptible individuals regarding the potential nephrotoxic risks of medications, which can lead to hyperfiltration, tubular and cortical toxicity, interstitial nephritis, crystalline nephropathy, and papillary necrosis. These include non-steroidal anti-inflammatory drugs (NSAIDs), antibiotics (including neomycin, kanamycin, paromomycin, bacitracin, polymyxin B, colistin, and amphotericin B), anticancer drugs, and more. Awareness about the nephrotoxic risks of off-label herbal substances such as licorice, aloe, and ephedra should also be raised [13].
4. *Continuous medical follow-up using accessible biochemical biomarkers*: Follow-up of patients living with systemic arterial hypertension should include evaluations of serum creatinine or cystatin C (where available) at the point of first evaluation and clinical care and with subsequent follow-up visits. Furthermore, all patients should be assessed for estimated GFR (eGFR) using the CKD-EPI 2021 equation and albuminuria (using nephelometry or turbidimetry techniques), as well as casual and morning urinary albumin-to-creatinine ratio assessments (ACR index). It is important to note that an eGFR < 60 mL/min/1.73 m2 and an ACR index > 30 mg/g are indicative of CKD-related damage in systemic arterial hypertension [14–16].
5. *Consider the nephroprotective role of antihypertensive drugs*: Systemic arterial hypertension-related kidney damage may be caused by several mechanisms, including vascular injury, hyperfiltration, and glomerular hypertension, amongst others. Because of its effects in improving endothelial dysfunction and reducing vascular injury, all antihypertensive drugs offer some level of nephroprotection. However, not all antihypertensive agents have positive and significant effects in ameliorating hyperfiltration and glomerular hypertension. Hyperfiltration depends on afferent arteriolar vasodilation and efferent vasoconstriction, which can result from impairments in the renin–angiotensin–aldosterone system (RAAS) often observed in diabetes, obesity, excess sodium, and protein intake. Angiotensin-converting enzyme (ACE) inhibitor and angiotensin II receptor blocker (ARBs) use leads to post-capillary, efferent arteriolar vasodilation, increasing glomerular flow without increasing glomerular pressure or filtration [17].

### Section 2: strategies for early detection of kidney disease

CKD is diagnosed when a decrease in eGFR < 60 ml/minute/1.73m^2^ is documented or with the onset of albuminuria ≥ 30 mg/g (3 mg/mmol), hematuria of glomerular-origin, kidney transplant, or any documented structural alteration through laboratory or imaging tests, which persists for at least three months [18]. CKD is a global public health issue, as it is associated with high mortality rates and is a significant risk factor for cardiovascular mortality. In Mexico, KD-related mortality currently stands amongst the top ten causes of death among individuals ≥ 45 years [19]. In 2021, there were 14,376 deaths due to kidney failure in Mexico, with CKD accounting for 71.8% (10,316 deaths) of these cases [20]. Additionally, CKD poses a significant economic challenge to the healthcare sector due to the prohibitive costs of treating end-stage KD (ESKD), its cardiovascular consequences, and the years lost due to premature mortality [21]. Therefore, early diagnosis and prevention of CKD and delaying its progression should be a priority to reduce complications and improve patient outcomes.

#### Question 2.1: does the determination of proteinuria have the same prognostic value for kidney function and the development of cardiovascular complications as albuminuria?

**Fig. 2:**
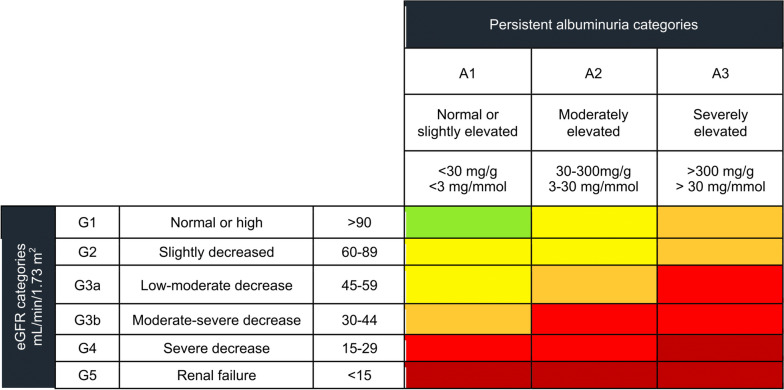
Staging and prognosis of chronic kidney disease according to estimated Glomerular Filtration Rate (eGFR) and albuminuria categories based on the KDIGO guidelines

Albuminuria is an independent risk factor for both all-cause and cardiovascular disease (CVD) mortality, and its detection should rely on quantitative measures such as the albumin-to-creatinine (ACR) ratio [22, 23]. As a crucial marker for progression to ESKD, identifying albuminuria as the earliest indicator of renal damage progression could be helpful in establishing therapeutic approaches which minimize the burden of adverse outcomes in patients living with systemic arterial hypertension. In the context of Mexico and Latin America, the following clinical strategies aim to estimate impaired kidney function based on albuminuria and eGFR:

1. *Determination of albuminuria and eGFR in all patients living with systemic arterial hypertension*: All patients with risk factors for developing CKD (> 60 years, diabetes, hypertension, overweight or obesity, previous CVD, use of nephrotoxic drugs, hereditary/familial history KD or hypertensive disease in pregnancy) should have frequent albuminuria and eGFR determinations [24]. Physicians should schedule follow-up visits and new evaluations, using albuminuria as the primary marker for predicting CKD progression, and refer patients to a nephrology specialist when necessary [25, 26]. In Fig. 2, we append the risk stratification of KD based on albuminuria and eGFR according to the KDIGO guidelines [24].
2. *Albuminuria and proteinuria are metrics of renal structural damage*: Albuminuria and endogenous proteinuria should not be used as equivalents to decreased kidney function, as they are not markers of kidney function but only indicators of structural damage within the kidney [27].
3. *Estimate the risk of CKD progression and CVD using the KDIGO calculator*: Although albuminuria is a strong predictor of CKD progression and CVD, some traditional risk estimations, such as the Framingham Risk Prediction Score, are not accurate in patients with CKD [28, 29]. To address this issue, KDIGO clinical guidelines have developed a CVD risk stratification score based on eGFR and albuminuria, which improves risk prediction, particularly given that the magnitude of albuminuria in CKD predicts a higher risk of CVD [24]. Additionally, this calculator estimates the probability of initiating renal replacement therapy (RRT) for patients with CKD in clinical practice (http://ckdpcrisk.org/lowgfrevents/) [30]. This KDIGO calculator has been developed for risk prediction of RRT, risk of death, and CVD in patients with CKD, using eGFR and albuminuria as two of the evaluated criteria.
4. *Use treatments based on ACE inhibitors or ARBs*: ACE inhibitors and ARBs can reduce initial albuminuria by 30–52%, preserving kidney function and decreasing the incidence of CVD events, which are the leading cause of death in patients living with CKD [31, 32]. However, controversy persists over whether reducing albuminuria or blood pressure as a single strategy can effectively reduce the risk of progression to CKD in patients living with systemic arterial hypertension.

#### Question 2.2: what is the ideal formula to estimate eGFR in non-dialysis patients?

The eGFR is an essential tool for diagnosing CKD and determining its prevalence, as it helps assess KD progression, CVD risk, and related complications. eGFR also guides decisions on drug limitations, dosing, RRT initiation, and certain procedures’ feasibility. While invasive methods can precisely determine eGFR, they are not routinely used in clinical practice. Instead, estimation equations based on serum creatinine levels and clinical data serve as the primary method for eGFR determination [33]. However, serum creatinine concentrations can vary depending on factors such as age, gender, muscle mass, and diet. The most widely used eGFR equations are the Cockcroft-Gault (CG), MDRD, and CKD-EPI equations, with the most recent 2021 equation removing the race coefficient [34–36]. These eGFR equations are critical in establishing CKD diagnoses and stratifying patients using the KDIGO guidelines, evaluating disease progression, and determining appropriate treatments. However, these equations may underestimate or overestimate CKD, depending on the clinical scenario and target population where they are incorporated [37]. In the context of Mexico and Latin America, the following strategies to estimate eGFR based on formulas are endorsed:

1. *Standardize plasma creatinine determinations*: It is necessary to standardize plasma creatinine determinations across different equipment and manufacturers to improve reproducibility and reduce classification errors, especially around 60 ml/min/1.73 m^2^ [38].
2. *Facilitate digital access to eGFR calculation tools*: Develop and promote electronic calculators that provide results for multiple equations (CG, MDRD, CKD-EPI 2009 and 2021) so that physicians can choose the most appropriate equation for each patient’s clinical context.
3. *Promote awareness and availability of cystatin C-based equations*: These approaches can be used as alternatives to creatinine-based equations, especially when creatinine production is abnormal (decreased muscle mass, diet, elderly, liver disease). Cystatin C could improve CKD diagnosis, screening, and monitoring and be a useful alternative in specific patient populations.
4. *Individualize the use of eGFR equations according to the patient’s needs*: Based on the individual patient’s characteristics, prioritize formulas that consider specific clinical scenarios, such as those in patients using anticoagulation medications or drugs that can modify creatinine clearance. Understand the limitations and benefits of each equation and apply them specifically [39].
5. *Specify the eGFR equation in algorithmic calculators*: Explicitly mention the equation used for eGFR estimation in different risk assessment tools, such as QRISK-SCORE 3 (cardiovascular risk calculator that considers CKD and requests eGFR with MDRD 4), MEHRAN SCORE (contrast-induced nephropathy risk calculator after PCI that requests eGFR with MDRD 4), and CRUSADE SCORE (hospital bleeding risk calculator after myocardial infarction that requests eGFR with Cockcroft-Gault).

Choosing the right eGFR equation is crucial for diagnosing and managing CKD. Standardizing creatinine measurements, facilitating digital access to eGFR calculators, conducting studies for local reference values, promoting alternative markers like cystatin C, and individualizing the use of eGFR formulas based on patient needs can improve CKD diagnosis and management in Mexico and Latin America.

#### Question 2.3: what is the recommended technique for detecting albuminuria in patients with systemic arterial hypertension?

Proteinuria, characterized by elevated levels of urinary proteins, is a marker for KD progression and is associated with CVD morbidity and mortality [40, 41]. In contrast, albuminuria enables earlier CKD diagnosis and assists in the extrarenal diagnosis of systemic complications. Current guidelines advise annual screening for CKD in at-risk populations by assessing eGFR and albuminuria using three determinations [24]. The 2012 KDIGO guideline for managing CKD recommends stratification based on the urinary ACR, preferably from the first-morning urine sample, as it strongly correlates with values measured in 24-h urine samples [18]. Furthermore, regular screening for urinary albumin is recommended for individuals living with systemic arterial hypertension based on the following factors:

1. When significant albuminuria is present when two out of three samples collected over at least three months show increased values.
2. When patients are diagnosed with CKD and albuminuria (ACR > 300 mg/g or > 30 mg/mmol), monitoring can be performed using the urinary protein/creatinine ratio (PR/CR).
3. The protein-to-creatinine in urine is recommended for patients with suspected renal interstitial pathology since proteinuria in these contexts stems from low molecular weight tubular proteins, distinct from albumin.

Accurate interpretation of results guides treatment response and prognosis, with changes in proteinuria/albuminuria levels serving as a “therapeutic target” and being associated with CKD progression or regression. Moreover, a reduction in ACR implies a decreased risk of needing RRT. As albuminuria is the earliest and most cost-effective marker of CKD, its use should be widely adopted in daily clinical practice for diagnosis, disease classification, therapeutic targeting, analysis of therapeutic outcomes (progression or regression), and prognosis for cardiovascular complications and the need for RRT [42, 43]. In the context of Mexico and Latin America, the following strategies for estimating albuminuria are proposed:

1. *Health personnel training is crucial for accurate interpretation*. Digital algorithms can be developed to guide the action based on ACR monitoring.
2. *Encourage all clinicians to estimate both eGFR and urine ACR*: This will provide primary care physicians with both diagnostic criteria for KD and allow subsequent studies to observe values over time, increasing KD diagnosis graphically. Confirming KD diagnosis will ensure timely patient referral to nephrology as these are strong predictors of mortality in patients living with CKD, even in the initial stages of the disease [44].
3. *Consider including albuminuria in primary cardiovascular risk scores*: This will have a direct impact on physician care, promoting early initiation of proteinuria-reducing treatments (e.g., ACEIs/ARBs, sodium-glucose cotransporter 2 inhibitors [SGLT2i]/ non-dihydropyridine calcium channel blockers [CCBs]).
4. *Monitor patients to assess the possibility of residual albuminuria:* Persistent albuminuria is defined as when albuminuria is present after six months of treatment with maximum pharmacological doses of ACEIs/ARBs, low-protein diet, and lipid-lowering agents). Residual albuminuria has been shown to have a linear relationship with subsequent risk of KD, just as initial, untreated albuminuria has a higher likelihood of progressing to CKD and maintaining elevated CVD risk [45].

In line with the strategies mentioned above, it is essential to develop a “guided search” for albuminuria when a patient at risk of developing CKD (e.g., patients with hypertension, type 2 diabetes, previous CVD, ≥ 60 years, obesity, patients on nephrotoxic drugs, etc.).

### Section 3: strategies for diagnose and monitor systemic arterial hypertension

Office-based blood pressure (BP) measurement continues to be a valid and useful systemic arterial hypertension screening and diagnosis method. However, out-of-office BP monitoring, including ambulatory BP monitoring (ABPM) and home BP monitoring (HBPM), provides alternative approaches that are linked to predicting systemic arterial hypertension-mediated organ damage, major CVD events, and mortality [46]. In the context of patients with CKD, BP monitoring poses a challenge due to masked systemic arterial hypertension, white-coat hypertension, and 24-h variability patterns at either ABPM or office-based BP measurements. Accurate monitoring and continuous vigilance of BP in systemic arterial hypertension are crucial for ensuring proper treatment and preventing progression to end-stage CKD. Therefore office-based BP measurements alone are insufficient, and it is essential to complement them with methods such as ABPM and/or HBPM in patients living with CKD.

#### Question 3.1: which of the different techniques for measuring BP outside the office is ideal for patients living with CKD?

##### CKD without dialysis

Achieving proper control and diagnosis of systemic arterial hypertension in patients living with CKD can be challenging. This is partly due to the high prevalence of conditions such as white coat hypertension, which affects approximately 30% of CKD patients submitted to RRT [47]. Additionally, masked systemic arterial hypertension is observed in 8–20% of the general population [48]. Cupisti et al. demonstrated the superiority of HBPM over office-based BP measurements in identifying the white coat effect in 62.5% of patients and masked systemic arterial hypertension in 22.7% of patients with stage 3–5 CKD [49]. Furthermore, the relationship between elevated BP and CVD is significant; notably, patients with high BP variability exhibit increased rates of CVD and mortality [50–52]. In a cohort of 1,219 patients living with CKD, high BP was independently correlated with organ damage [53]; similarly, Drawz et al., also demonstrated in a cohort of 1,492 patients with CKD an independent association between masked systemic arterial hypertension and end-organ damage [54]. Consequently, the association between uncontrolled high BP and clinical outcomes in patients with systemic arterial hypertension and CKD is significant.

In patients with CKD who are not receiving RRT, a stronger correlation has been observed between HBPM or ABPM and a decline in eGFR and/or need for RRT, compared to office-based BP measurements [55]. Moreover, ABPM outperformed office-based measurements in predicting composite events of all-cause mortality and the need for RRT, though it did not demonstrate superiority over HBPM [56]. In a cohort study by Borelli et al., which included 906 CKD patients, factors most strongly associated with an increased risk of fatal and non-fatal CVD events and kidney function deterioration were the absence of nocturnal dipping and systolic BP exceeding the ABPM target (> 135 mmHg during the day or > 120 mmHg at night) [57]. Consequently, for CKD patients without dialysis, ABPM is the recommended method for BP measurement.

##### CKD on dialysis

Diagnosing and monitoring systemic arterial hypertension in patients undergoing RRT, particularly hemodialysis (HD), is a considerable challenge. Mansoor and White reported that almost 80% of adults on HD were classified with systemic arterial hypertension, yet only 30% achieved BP goals [58]. Additionally, studies have shown that when a 44-h interdialytic ABPM was conducted, only 33% of the population met the criteria for systemic arterial hypertension, highlighting the difficulty of managing systemic arterial hypertension in RRT settings. In patients submitted to dialysis, nighttime BP measurements strongly predict overall outcomes [59]; a study performed by Tripepi et al. identified the night/day BP ratio as a prognostic factor for clinical outcomes in these patients [60]. Similarly, Ekart R. et al. described that 48-h ABPM, rather than office-based BP measurements, were independent predictors of CVD death in HD patients [61]. Finally, Alborzi et al. associated HBPM with CVD mortality and all-cause mortality [62]. Therefore, both night/day BP ratios and HBPM are superior predictors of CVD events compared to office-based or peridialytic period BP measurements. These findings emphasize the importance of utilizing appropriate diagnostic and monitoring tools for managing systemic arterial hypertension in patients on RRT.

For patients with systemic arterial hypertension and CKD, substantial evidence supports the superiority of both ABPM and HBPM over office-based BP measurement in accurately diagnosing systemic arterial hypertension, predicting major CVD events, kidney function decline, and CVD and all-cause mortality. Considering the context of Mexico and Latin America, the following strategies for out-of-office BP measurement in patients with CKD are proposed:

1. *ABPM should be the method for diagnosing systemic arterial hypertension*: Consistent with existing evidence, ABPM offers relevant prognostic value due to its ability to assess nighttime compared to daytime BP. Moreover, ABPM has the most significant impact on a patient’s clinical prognosis in the context of CKD.
2. *If ABPM is unavailable, HBPM is an excellent alternative*: HBPM is a superior option for identifying masked and white coat arterial hypertension, as it demonstrates a better correlation with CVD, KD, and mortality compared to office-based BP measurements.
3. *Office-based BP measurement should be used for screening and detecting systemic arterial hypertension:* Given the evidence highlighting the superiority of both ABPM and HBPM for accurate diagnosis and prognosis, office-based BP measurements should be primarily employed for screening in patients with CKD and systemic arterial hypertension.

### Section 4: strategies to achieve BP Targets in patients living with CKD

In patients living with CKD, systemic arterial hypertension prevalence increases with KD progression, reaching 90% prevalence in patients classified with CKD stage 5 [63]. The key mechanisms related to systemic arterial hypertension in patients living with CKD include volume overload, sympathetic hyperactivity, salt retention, endothelial dysfunction, and alterations in BP-regulating hormone systems [64]. Moreover, in kidney transplant recipients, systemic arterial hypertension etiology is multifactorial, depending on the recipient and donor characteristics and transplant-specific causes (e.g., immunosuppressive medications, allograft dysfunction, and surgical complications like transplant artery stenosis) [65]. High BP is an independent risk factor for CVD morbidity-mortality and adverse KD outcomes, making BP control a crucial goal for adequate clinical management. However, optimal BP goals in hypertensive CKD patients, specifically kidney transplant recipients, remain controversial [66].

#### Question 4.1: what are the risks/benefits of key-strategies in patients living with CKD who survive stage 3 KDIGO?

##### Target BP in patients with CKD without dialysis

The ideal BP targets in patients with CKD without dialysis vary widely across systemic arterial hypertension guidelines. The American College of Cardiology/American Heart Association (ACC/AHA) recommends a BP target of < 130/80 mmHg for stage 5 CKD patients. This goes in line with threshold recommendations by the European Society of Cardiology/European Society of Hypertension (ESC/ESH), which also suggest a BP target < 130–139 mmHg for the same population [67, 68]. Conversely, the updated National Institute for Health and Care Excellence (NICE) guidelines recommend a BP target of < 140/90 mmHg for patients with ACR < 70 mmol/mol and systolic BP (SBP) of 130 mmHg and BP < 130/80 mmHg (considering lower SBP of 120 mmHg) for those with CKD and ACR > 70 mmol/mol [69].

The 2021 KDIGO Clinical Practice Guideline for BP management in CKD recommends treating patients not receiving RRT using an SBP threshold of < 120 mmHg [70]. This strategy is primarily based on the SPRINT study subgroup analysis of CKD patients, which demonstrated a 25% risk reduction in major adverse CVD events using this threshold. However, for patients with eGFR < 45 ml/min/1.73m^2^, no reduction in CVD risk was observed with these SBP targets, although reductions in all-cause mortality and mild cognitive impairment were observed [71, 72]. Nevertheless, it has been further questioned whether an SBP target < 120 mmHg is the ideal threshold as it has been associated with increased risks of adverse outcomes, including hypotension, syncope, electrolyte abnormalities, and acute kidney injury or failure. A sub-analysis of the ACCORD trial compared intensive SBP control (SBP < 120 mmHg) with standard control (SBP < 140 mmHg) in patients living with diabetes and found no overall benefit of intensive BP control, except for a reduced risk of non-fatal stroke [73]. Recently, three systematic reviews and meta-analyses examined the benefits of intensive BP control in patients living with CKD. The first meta-analysis performed by Tsai et al. found no additional benefit of intensive BP control compared to standard control on KD outcomes [74]. Conversely, a second meta-analysis showed that intensive BP reduction was associated with significantly lower mortality risk compared to less intensive BP control [75]. The last meta-analysis found no differences in death, cardiovascular death, or heart failure when comparing various SBP targets. However, lower BP targets were associated with higher adverse effects at follow-up [76]. Based on this evidence, we suggest that patients living with CKD without dialysis should have SBP targets of < 130 mmHg, as this threshold could lead to an optimal balance between efficacy and safety.

##### Target blood pressure in patients with CKD with dialysis

The 2021 KDIGO Guideline recommends a BP target of < 130/80 mmHg for kidney transplant recipients [70]. This recommendation is based on expert opinion, as no clinical trials have investigated the relationship between this BP target and adverse clinical outcomes. Nevertheless, it has been reported that lower BP has been associated with eGFR decline, CKD progression, and acute kidney injury events in kidney transplant recipients [76]. A more conservative target is suggested, as kidney transplant recipients with a single kidney may be more vulnerable to these adverse effects. This recommendation aligns with the 2017 ACC/AHA guideline on hypertension, which also proposes a BP target of ≤ 130/80 mmHg for kidney transplant recipients [68].

Considering the context of Mexico and Latin America, the following strategies for BP goals in patients with CKD are proposed:

1. *Adopt standardized BP measurement according to KDIGO guidelines: *This strategy is based on ensuring accurate and consistent BP readings for patients living with CKD, considering factors such as resting, avoiding caffeine, exercising, and smoking before BP measurement.
2. *Address challenges in performing standardized BP measurements in clinical practice*: Due to time constraints and institution-specific clinical policies, it may be difficult to perform standardized BP measurements; however, it is essential to prioritize accurate BP readings for patients living with CKD.
3. *Recognize the white coat effect on office BP readings*: Non-standardized office BP readings can be significantly higher than standardized BP measurements, which can affect treatment decisions for patients living with CKD. In this context, home BP monitoring can help to eliminate the “white coat” effect by providing a more accurate assessment of BP levels.
4. *Be cautious when applying SBP targets to non-standardized BP measurements*: As previously discussed, applying an SBP target of < 120 mmHg can lead to overtreatment and increased adverse events in patients living with CKD. Therefore, most patients living with CKD should achieve and maintain BP levels < 130/80 mmHg as this target ensures a well-tolerated BP for most patients [77].
5. *Implement improved BP measurement methods in clinical settings*: Creating specialized areas for standardized BP measurement and employing trained nursing staff can ensure accurate BP readings for patients with CKD in clinical settings [78].
6. *Involve health authorities in promoting health goals and continuous training*: Health authorities should support the achievement of health goals, reduce long-term expenditure, and provide continuous training for medical professionals to ensure the best possible care for patients living with CKD.

Given the lack of strong evidence, the current SBP target of < 120 mmHg for patients with CKD recommended by KDIGO is inappropriate for most patients and may even be harmful to those with CKD stages 4 and 5, diabetes, glomerulopathies, polycystic KD, and proteinuria. Based on previous evidence, we endorse an SBP target of < 130/80 as this goal is more reasonable and well tolerated.

### Section 5: hypertensive treatment strategies in patients living with CKD

CVD is the leading cause of mortality in patients living with CKD in Mexico [79]. Consequently, BP control is the main pillar to mitigate the burden of complications related to systemic arterial hypertension and CKD. Nevertheless, several risk factors have been found to be associated with difficult-to-control BP, including albuminuria, volume overload, and sympathetic nervous system hyperactivity with circadian rhythm alterations [79]. As kidney function declines, cardiovascular health deteriorates, and the risk of adverse outcomes increases [80, 81]. Therefore, cardio-nephroprotection from the early stages of KD is crucial. Current guidelines propose that managing systemic arterial hypertension in patients with CKD should begin with RAAS inhibitors due to their demonstrated efficacy in reducing various markers of KD progression. Second-line drugs, such as CCBs, have also positively affected albuminuria, eGFR preservation, and circadian rhythm BP variability. However, there are specific considerations when using RAAS inhibitors and CCBs in clinical practice.

#### Question 5.1: can we expect the same results on CKD progression and CVD outcomes for all RAAS inhibitors?

##### ACEIs and ARBs

ACEIs and ARBs are indispensable for treating systemic arterial hypertension in patients living with CKD [81]. Their use is also well-established in treating albuminuria, heart failure with reduced ejection fraction, and acute myocardial infarction. In Table 1, we present the results of two primary meta-analyses comparing different pharmacological groups of RAAS inhibitors and their clinical use for adverse outcomes. Both studies found that using ACEIs and ARBs in patients living with CKD reduces the risk of renal failure and CVD events. Additionally, ACEIs have been shown to decrease all-cause mortality and may be superior to ARBs in reducing CVD death. This suggests that ACEIs could be the first therapeutic option for this population [82]. However, some authors, such as Strippoli et al., concluded in a Cochrane systematic review that there are no significant differences in overall mortality when comparing ACEIs versus ARBs [83]. The debate continues, but the main strategy emphasize using the maximum tolerable dose of ACEIs to optimize and manage CKD-related outcomes.

**Table 1:** Studies comparing the effect of ARBs and ACE inhibitors for kidney disease and cardiovascular outcomes in patients with chronic kidney disease

Study	Evaluated Outcomes	Results
Trialists collaboration (Meta-analysis of 119 RCTs with ACE inhibitors and ARBs)	Kidney Disease and Cardiovascular Outcomes in patients with CKD	• ACE inhibitors reduce risk of kidney failure by 39% and ARBs by 30% (OR 0.61 (95%CI 0.47–0.79, OR 0.70 95%CI 0.52–0.89) respectively compared to placebo. ACE inhibitors and RBs reduced risk of cardiovascular outcomes by 35% and 25% respectively compared to active controls, while the active controls did not show significant evidence of renal benefits. • Both ACE inhibitors and ARBs reduce the likelihood of major cardiovascular events with OR 0.82 (95%CI 0.71–0.92) and 0.76 (95%CI 0.62–0.89) respectively versus placebo. ACE inhibitors but not ARBs showed a significant reduction in all-cause mortality compared to active controls (OR 0.72 95%CI 0.53–0.92).
Strippoli et al (Cochrane Systematic Review of 49 studies with 12,067 patients)	All-cause mortality and Kidney Disease Outcomes (progression of albuminuria)	• There was no significant difference in all-cause mortality for ACE inhibitors versus placebo (RR 0.91, 95%CI 0.71 to 1.17) or ARBs versus placebo (RR 0.99, 95%CI 0.85 to 1.17). • A reduction in all-cause mortality risk was found with maximum tolerable doses of ACE inhibitors compared to half-doses (RR 0.78, 95%CI 0.61 to 0.98). • Mortality was similar in studies with ACE inhibitors and ARBs. Renal effects (prevention of albuminuria progression) were similar between groups.

*Abbreviations*: *RCT* Randomized Clinical Trial, *ACE* Angiotensin Converting Enzyme, *ARB* Angiotensin II Receptor Blockers, *OR* Odds Ratio, *RR* Risk Ratio

##### Calcium channel blockers

CCBs play a key role in treating systemic arterial hypertension in patients living with CKD. The main evidence stems primarily from the ACCOMPLISH study, which found that the benazepril-amlodipine combination was superior to benazepril-hydrochlorothiazide in reducing CVD events and slowing CKD progression [84]. Subsequently, several meta-analyses and systematic reviews have been conducted to clarify the potential benefits of CCBs. The primary results of three meta-analyses are presented in Table 2. The first study, conducted by Fu et al., provides evidence that initiating RAAS inhibitors compared to CCBs offers renal benefits in patients with CKD and similar cardiovascular protection [85]. This study shows that the benefits continue with ACEIs and ARBs in ESKD patients. Another study by Lin et al. found a similar long-term effect on BP control, mortality, heart failure, stroke events, and kidney function in patients with stage 3–5 CKD with systemic arterial hypertension using CCBs and RAAS inhibitors [86]. Finally, Zhao et al. conclude that the best therapeutic option combines RAAS inhibitors with CCB [87]. In summary, using CCBs is an excellent therapeutic option when combined with another RAAS inhibitor. In the case of patients where ACEIs/ARBs are not well-tolerated, non-dihydropyridines CCB (e.g., Diltiazem) have been demonstrated to have beneficial effects on BP management and the time course of blood pressure [88, 89].

**Table 2:** Studies comparing the effect of RAAS inhibitors drugs versus CCBs in renal and cardiovascular outcomes in patients with kidney disease

Study	Evaluated Outcomes	Results
Fu et al (Effectiveness of RAAS inhibitors vs CCBs)	Risk of initiation of renal replacement therapy (RRT), all-cause mortality, and cardiovascular events in older patients with advanced CKD in a 10-year cohort study	• Significantly lower risk of RRT initiation following new use of ACE inhibitors compared to new use of CCBs (adjusted HR, 0.79 [95%CI, 0.69–0.89]), but similar risks of mortality (adjusted HR, 0.97 [95%CI, 0.88–1.07]) and MACE (adjusted HR, 1.00 [95%CI, 0.88–1.15]). • The positive control cohort of patients with G3 CKD showed a similar reduction in the risk of RRT initiation (adjusted HR, 0.67 [95%CI, 0.56–0.80]) with ACE inhibitor therapy compared to CCBs.
Lin et al (Systematic Review and Meta-Analysis comparing CCBs and RAAS inhibitors in patients with SAH and stage 3–5 CKD)	Changes in blood pressure, all-cause mortality, heart failure, cerebrovascular events, and renal outcomes.	• 21 studies with 9492 patients were analyzed and no significant differences were observed in any observed outcome.
Zhao et al (Systematic Review and Meta-analysis)	8 clinical trials with 25,647 patients were analyzed, examining the effect of CCBs on the incidence of CKD and all-cause mortality compared to ACE inhibitors and ARBs	• Decrease in blood pressure was similar for all pharmacological groups, with no significant difference in all-cause mortality, but finding a better nephroprotective effect for ACE inhibitors and ARBs compared to CCBs (IRR 0.96, 95%CI, 0.89–1.03).

*Abbreviations*: *RAAS* Renin-Angiotensin Aldosterone System, *CCB* Calcium Channel Blockers, *CKD* Chronic Kidney Disease, *SAH* Systemic Arteria Hypertension, *MACE* Major Cardiovascular Events, *ACE* Angiotensin Converting Enzyme, *IRR* Incidence Rate Ratio, *HR* Hazard Ratio

#### Question 5.2: what are the safe therapeutic strategies to reduce BP and albuminuria, and control the circadian rhythm BP variability in stage 4–5 CKD?

##### Albuminuria

Pharmacological RAAS blockade using ACEIs, and ARBs is still the treatment of choice for reducing albuminuria, as showed by Lambers et al. in a meta-analysis examining the effects of pharmacological proteinuria reduction and nephroprotection. The analysis primarily included studies with RAAS inhibitors, which showed an average albuminuria reduction of 19.2% and significant differences compared to the control group. A crucial association was found, showing that for every 30% reduction in albuminuria, the risk of end-stage KD decreases by 23.7% [90]. In this context, new evidence has emerged about the benefits of reducing albuminuria linked to the use of CCBs. In a study by Abraham et al., they suggest that dihydropyridine CCBs can be used as a first-line treatment in non-proteinuric CKD, but when combined with RAAS inhibitors, they have a more significant impact on proteinuric CKD [91]. Additionally, CCBs that act on L/N and L/T channels also have a substantial effect on decreasing albuminuria and intraglomerular pressure, improving renal hemodynamics, and producing a more significant reduction in proteinuria, even in patients already treated with RAAS inhibitors. Additionally, CCBs inhibit aldosterone secretion, improve endothelial dysfunction, and reduce oxidative stress [92]. An association between CCBs and an increase in the glomerular filtration rate has been observed, thereby delaying the onset of KD. Consequently, CCBs could present an alternative for treating systemic arterial hypertension in patients living with CKD, although more long-term studies are needed to confirm its impact on clinical outcomes.

##### Sodium-glucose cotransporter 2 inhibitors

A new pharmacological group of interest for cardio-renal protection and reduction of albuminuria are SGLT2i. Developed for the treatment of type 2 diabetes, SGLT2i works by inhibiting proximal glucose reabsorption. Additionally, recent evidence has discovered the pleiotropic effects of SGLT2i, such as natriuresis, intravascular volume contraction, and intra-renal hemodynamics modification, contributing to the beneficial effects on BP, body weight, and albuminuria. Evidence from the EMPA-REG and studies show a reduction in adverse CVD events with the use of SGLT2i compared to placebo [93, 94]. Similarly, the CREDENCE study found a significant reduction in albuminuria and CVD events with SGLT2i [95]. A substantial reduction in adverse events is evident in the decrease of heart failure and progression of KD with SGLT2i. To evaluate outcomes in patients with stage 3–4 CKD, Li et al. conducted a meta-analysis and found that the use of SGLT2i effectively reduced the risk of primary CVD outcomes by 26%, with reductions of 30% in patients with stage 3a CKD, 23% in stage 3b CKD, and 29% in stage 4 CKD. The results were similar for the reduction of heart failure with reduced ejection fraction in patients with KD and patients with arteriosclerosis [96]. This evidence shows the increasing potential benefits of adding SGLT2i for decreasing the progression and managing systemic arterial hypertension in patients living with CKD.

##### Circadian rhythm BP variability

CCBs are useful for controlling circadian rhythm BP variability. The action time of dihydropyridine CCBs, such as amlodipine, has been seen to control BP for 24 h, thereby minimizing BP variability. This decrease in variability results in a reduction of kidney function deterioration and CVD complications [91]. The ASCOT-BPLA (Anglo-Scandinavian Cardiac Outcomes Trial-Blood Pressure Lowering Arm) study evaluated the treatment with CCBs in combination with or without ACEI, beta-blockers, and diuretics [97]. The findings revealed that treatment with amlodipine was superior to beta-blockers, showing a reduction in BP variability and prevention of CVD events.

##### Risk of hyperkalemia and kidney function deterioration

Although it is well established that using RAAS inhibitors slows the progression of CKD, some studies suggest discontinuing it when CKD advances due to potential kidney function deterioration. Bhandari and colleagues published a multicenter study and found no differences between the control group and the group using RAAS inhibitors in terms of KD outcomes. The study concluded that discontinuing RAAS inhibitors is not associated with a decline in eGFR in the long term [98].

In summary, ACEI and ARBs have similar effectiveness in managing KD and CVD outcomes in CKD patients, with a preference for ACEI. Combining a RAAS inhibitor with a dihydropyridine CCB is suggested for optimal results. Controlling albuminuria and BP variability is essential, for which SGLT2i shows promising results in reducing CVD and KD adverse events. Nevertheless, there is a need for affordable and accessible medications to improve treatment adherence and implementation of multidisciplinary management, which are crucial for effectively treating patients living with CKD.

### Section 6: strategies of usage of diuretics and non-steroidal mineralocorticoid receptor inhibitors in patients with CKD

For patients with CKD, thiazide and loop diuretics are highly recommended. Thiazides lower BP by increasing urinary sodium excretion, which leads to a decrease in extracellular volume. Thiazides have been proven to improve CVD outcomes, including stroke, heart failure, coronary events, and CVD death. When used in combination with other pharmacological groups, such as ACEI, ARBs, and CCBs, thiazides enhance antihypertensive and cardioprotective effects [99]. Conversely, for patients with CKD and eGFR < 30 ml/min/1.73 m^2^, the use of thiazide has shown less effectiveness [100]. In these patients, loop diuretics positively impact reducing volume overload and improving functional class, although their antihypertensive effects are limited [101]. Diuretics should be avoided in patients with CKD secondary to polycystic KD, as they increase cyst size and promote the loss of tubular excretory function [102].

#### Question 6.1: what is the utility of Diuretics and Non-Steroidal Mineralocorticoid Receptor Antagonists (nsMRAs) in treating systemic arterial hypertension with CKD?

##### Mineralocorticoid receptor antagonists

The RAAS plays a crucial role in BP control, extracellular fluid volume regulation, and serum potassium levels. Persistent activation of mineralocorticoid receptors significantly affects cardiomyocytes, fibroblasts, podocytes, and endothelial cells, leading to ventricular hypertrophy, fibrosis, and proinflammatory activity [103]. The first MRA, spironolactone, was a non-selective agent. Highly selective nsMRAs such as finerenone, esaxerenone, and apararenone are available as nsMRAs have demonstrated better anti-inflammatory and antifibrotic activity without significant antihypertensive effects [104].

nsMRAs are effective in managing resistant systemic arterial hypertension, which is uncontrolled hypertension, despite full-dose use of three or more antihypertensive drugs, including a diuretic [105]. These antagonists carry the risk of causing hyperkalemia or reversible kidney function impairment, primarily in those with an eGFR < 30 ml/min/1.73 m^2^. neMRAs have shown benefits in the cardiorenal sphere; the FIDELIO study documented mild to moderate antihypertensive effects and an increased risk of hyperkalemia. In controlled clinical trials involving patients with type 2 diabetes mellitus, CKD, and systemic arterial hypertension, finerenone significantly reduces albuminuria [106].

##### New strategies for hyperkalemia management

Hyperkalemia is one of the most frequent complications in the context of RAAS inhibitors used in patients living with CKD. Various results show that nsMRAs such as finerenone pose a lower risk of hyperkalemia due to the drug’s distribution in the heart and kidney, compared to steroidal MARs (spironolactone and eplerenone) [107]. Risk factors for hyperkalemia include decreased eGFR combined with RAAS inhibitor use. Other risk factors include heart failure, diabetes mellitus, resistant systemic arterial hypertension, acute myocardial infarction, and drugs (heparin, NSAIDs, trimethoprim, and pentamidine).

Therapeutic approaches for patients with hyperkalemia begin with identifying risk factors and periodically checking potassium levels. The frequency of monitoring depends on the patient’s comorbidities and the combination of the mentioned risk factors. Persistent hyperkalemia management includes loop diuretics and thiazides, changing the dose of RAAS inhibitors, and finding enhancing drugs. However, RAAS-modulating drugs are essential in patients with CVD, predisposing them to hyperkalemia. In this circumstance, potassium-binding agents can benefit by limiting hyperkalemia [108, 109]. Potassium-binding drugs prevent intestinal potassium absorption and promote fecal excretion. The most commonly used molecules are kayexalate, patiromer, and sodium zirconium cyclosilicate. Initiating these pharmacological agents could be considered in patients with persistent hyperkalemia. Patiromer’s utility was proven in controlled clinical trials involving patients with hyperkalemia, CKD, and heart failure under treatment with RAAS inhibitors [110]. In these patients, escalating oral doses of patiromer between 8.4 g and 25.2 g every 12 h managed to attenuate serum potassium levels by up to 1 mEq/L. Sodium zirconium cyclosilicate is a recently approved potassium-binding agent for patients with chronic hyperkalemia. Clinical studies of its effectiveness come from patients with CKD, heart failure, and/or diabetes who have needed RAAS-modulating drugs. These studies have shown the utility of 5–10 g per day, with a decrease in potassium levels within the first 48 h [111].

## Conclusion

In conclusion, in this narrative review, we extensively analyzed strategies endorsed by the Mexican Group of Experts on Arterial Hypertension to prevent, diagnose, and treat CKD related to systemic arterial hypertension within the Mexican and Latin American context. Our review underscores the importance of multidisciplinary management of systemic arterial hypertension and the crucial role community-based programs play in improving the quality of life for affected individuals. By providing these tailored strategies, we aim to reduce the burden of systemic arterial hypertension related to CKD and its complications in Mexico and Latin America, ultimately benefiting millions of people across the region. Future efforts should be directed towards continuously evaluating and updating these strategies, incorporating new evidence as it emerges, and promoting their dissemination and implementation in healthcare settings throughout the region. In addition, further research should focus on identifying and addressing barriers to adopting these strategies and evaluating their real-world effectiveness in improving patient outcomes and reducing the impact of systemic arterial hypertension related to CKD in Mexico and Latin America.

## Data Availability

Not applicable.

## Abbreviations

ABPM: Ambulatory BP Monitoring
ACC/AHA: American College of Cardiology/American Heart Association
ACR: Albumin-to-Creatinine
AICEs: Angiotensin-Converting Enzyme
ARBs: Angiotensin II Receptor Blocker
BP: Blood Pressure
CCBs: Calcium Channel blockers
CG: Cockcroft-Gault
CKD-EPI: Chronic Kidney Disease Epidemiology Collaboration
CKD: Chronic Kidney Disease
CVD: Cardiovascular Disease
eGFR: Estimated Glomerular Filtration Rate
ESC/ESH: European Society of Cardiology/European Society of Hypertension
ESKD: End-Stage Kidney Disease
HBPM: Home Blood Pressure Monitoring
HD: Hemodialysis
KD: Kidney Disease
KDIGO: Kidney Disease: Improving Global Outcomes
MDRD: Modification of Diet in Renal Disease
nsMRAs: Non-Steroidal Mineralocorticoid Receptor Antagonists
NICE: National Institute for Health and Care Excellence
NSAID: Non-Steroidal Anti-Inflammatory Drugs
RAAS: Renin–Angiotensin–Aldosterone System
RRT: Renal Replacement Therapy
SBP: Systolic Blood Pressure
SGLT2i: Sodium-glucose Cotransporter-2 Inhibitors
